# Supplementary material for: Assessing the availability and scope of routine data on post-pregnancy family planning: A cross-sectional review of registers and reporting tools in 18 low- and middle-income countries
Source: PLOS Glob Public Health. 2025 Oct 9;5(10):e0005205. doi: 10.1371/journal.pgph.0005205 (PMC12510596; doi:10.1371/journal.pgph.0005205)
Supplement: Attachment — Submitted filename: Manuscript Review-PGPH-D-25-01121-MK.docx [file pgph.0005205.s001.docx]

**Manuscript PGPH-D-25-01121: Assessing the availability and scope of routine data on post-pregnancy family planning: a cross-sectional review of registers and reporting tools in 18 low- and middle-income countries**

**General comments -** The manuscript provides a good recent analysis of the status on availability of key PPFP data elements that are included in registers and monthly summary forms used for routine data collection in select LMICs.

**Specific Comments**

**Abstract** – The abstract is well written and summarizes the rationale for the study, methodology, key findings, and conclusions of the study.

**Introduction** - This section provides relevant background and rationale for the review. The authors have cited relevant indicators, initiatives geared at advancing availability or PPFP data and timelines, focusing on the focus of the manuscript.

**Materials and Methods** –**General:** While the description of the process followed to obtain the tools and reporting form from the various countries has been described well, additional details would be helpful for anyone planning to conduct a similar review in another or related health area. The authors should consider adding:

- Ensure all the acronyms are defined as some readers may not be familiar with them, e.g. MOMENTUM, R4S/SMART-HIPs
- Some registers might have been availed in electronic format (hopefully the majority). Include how tools/registers available in paper format ONLY were they handled (e.g. scanned, translated, variables typed by investigator in Ms Excell/Word document…etc.).
- Provide some more details on the analysis process, e.g. if any categories of data elements were generated, harmonization of “meanings” if different terms were used across different countries, how data were summarized and presented, etc.
- Text in rows 170-173 seem best suited for the “methods” section explaining how analysis, presentation and interpretation of findings was done; move from the results section.

**Results** – Findings presented well in logical subheadings making it easy to follow.

**Discussion –** This section can be improved/ strengthen by adding evidence based on use of longitudinal registers as opposed to cross-sectional registers to avoid overcounting / duplication (Refer to lines 299-304), especially if a woman receives multiple services in the same health facility.

**References** – Relevant and recent.
