# Supplementary material for: Assessing the availability and scope of routine data on post-pregnancy family planning: A cross-sectional review of registers and reporting tools in 18 low- and middle-income countries
Source: PLOS Glob Public Health. 2025 Oct 9;5(10):e0005205. doi: 10.1371/journal.pgph.0005205 (PMC12510596; doi:10.1371/journal.pgph.0005205)
Supplement: Attachment — Submitted filename: Response to reviewers_13Jul2018_PGPH-D-25-01121.docx [file pgph.0005205.s003.docx]

**RE:** Manuscript PGPH-D-25-01121: *Assessing the availability and scope of routine data on post-pregnancy family planning: a cross-sectional review of registers and reporting tools in 18 low- and middle-income countries*

Thank you to the editor and reviewers for the thoughtful feedback. We have made extensive changes to the paper based on the comments. Below is a summary of our response to each comment.

**Journal Requirements:**

Please review your reference list to ensure that it is complete and correct. If you have cited papers that have been retracted, please include the rationale for doing so in the manuscript text, or remove these references and replace them with relevant current references. Any changes to the reference list should be mentioned in the rebuttal letter that accompanies your revised manuscript. If you need to cite a retracted article, indicate the article’s retracted status in the References list and also include a citation and full reference for the retraction notice.

Reference list is complete and correct.

**Reviewer 1**

**General comments -** The manuscript provides a good recent analysis of the status on availability of key PPFP data elements that are included in registers and monthly summary forms used for routine data collection in select LMICs.

Thank you.

**Specific Comments**

**Abstract** – The abstract is well written and summarizes the rationale for the study, methodology, key findings, and conclusions of the study.

Thank you.

**Introduction** - This section provides relevant background and rationale for the review. The authors have cited relevant indicators, initiatives geared at advancing availability or PPFP data and timelines, focusing on the focus of the manuscript.

Thank you.

**Materials and Methods** –**General:** While the description of the process followed to obtain the tools and reporting form from the various countries has been described well, additional details would be helpful for anyone planning to conduct a similar review in another or related health area. The authors should consider adding:

- Ensure all the acronyms are defined as some readers may not be familiar with them, e.g. MOMENTUM, R4S/SMART-HIPs

MOMENTUM, though capitalized, is not an acronym. We checked other acronyms to ensure we defined them the first time used.

- Some registers might have been availed in electronic format (hopefully the majority). Include how tools/registers available in paper format ONLY were they handled (e.g. scanned, translated, variables typed by investigator in Ms Excell/Word document…etc.).

We added a sentence to explain the types of files we obtained.

- Provide some more details on the analysis process, e.g. if any categories of data elements were generated, harmonization of “meanings” if different terms were used across different countries, how data were summarized and presented, etc.

We have added details on the analysis process.

- Text in rows 170-173 seem best suited for the “methods” section explaining how analysis, presentation and interpretation of findings was done; move from the results section.

The information on how we handled the tools from 4 provinces in Pakistan was moved to the methods section.

**Results** – Findings presented well in logical subheadings making it easy to follow.

Thank you.

**Discussion –** This section can be improved/ strengthen by adding evidence based on use of longitudinal registers as opposed to cross-sectional registers to avoid overcounting / duplication (Refer to lines 299-304), especially if a woman receives multiple services in the same health facility.

Longitudinal registers or electronic health records have the potential to reduce counting the same client more than once, but these types of tools are not often used in developing countries and we do not feel that we can comment on their impact on PPFP data quality in this paper as it was not considered as part of the review.

**References** – Relevant and recent.

Thank you.

**Reviewer 2:**

Reviewer #2: I - This is an interesting piece of work on postnatal care data systems in selected LMICs, which is an area which remains underreported. There is a lot of work that has gone into the analysis of the tools, which is a merit of the manuscript. However, I have some concerns over the methodology and write-up, which require attention from the Authors. This is mainly on differentiating the work done as part of this study from previous work conducted, including stronger rationale for conducting the work given the previous reviews and the fact that some of the tools have not changed in the interim period.

We have added information on the limitations of the prior reviews and more rationale for why the current one was needed. This information was added to the introduction and discussion.

Second main concern is over the methodology and limitations, which although acknowledged, do not seem to have been taken into account when setting up the study and therefore limiting the robustness of the work. Strengthening the discussion and recommendations for researchers, practitioners and policy-makers requires attention to ensure those are more specific and underpinned by evidence from the work conducted.

Points well taken. We revised the limitations to add more information on what we did to mitigate them. We also revised the recommendations to ensure they are more specific and underpinned by evidence from this review.

Below are more details:
1. Firstly, in terms of the premise of the work covered:
- The focus is on tools covering registers and reporting tools, but there is limited insight into how those reflect the national policy. Specifically, Indicators for FP an PNC - what is the national guidance on provision and reporting? This may help to explain why indicators are or are not present and if present why they are phrased in a particular manner. This requires investigation.

Unfortunately, collecting and reviewing policy documents from each of the 18 countries is beyond the scope of what these authors can do in this paper, given each country has multiple policy documents to guide FP provision and reporting. The main point of the paper is not to understand if each country is responding to their own priorities, but to understand what we are seeing in data availability across countries. We made revisions to clarify the purpose of the paper.

- Similarly in terms of reporting, requirements at national and regional level, facility levels for registers and additional reporting tools are of relevance to the topic, yet the nuances not captured here. This needs attention.

All the data elements extracted from country tools are required at national level (or provincial level in the case of Pakistan).

- In addition to all the mentioned registers and reporting tools, there are patient-held cards that also include information on PNC FP and related services - this does not feature as a point in the manuscript, but may need some attention.

We did not review patient-held cards because they are used by providers to facilitate care to the patient, but information recorded in the cards is not aggregated and thus not available for program accountability. We added an explanation for why patient-held cards were excluded in the methods section.

- Indicators: this is a point that may need expanding to make this work more easily translatable into practice - based on your review, what indicators are used that show good practice? What are the gaps? (some of this features in the discussion but is fairly broad and not supported by evidence so needs expanding). In terms of reporting - again, how does your review help to see what works and what needs improving?

We have added more information to the discussion.

- Denominators: this is mentioned as a limitation, but really is an integral part of indicators and usability of data and therefore requires insights from the Authors.

We revised the limitations section to explain why we did not assess the denominators.

2. Secondly, there are aspects of the methods which potentially limit the usability of the work and require addressing and possible amending as part of revisions:
- In particular, the manuscript requires clear differentiation between what has been done, esp in light of dates of publication of tools used also in this review - how much of this work is duplication, how much is new needs to be more clearly delineated to show evidence of contribution to knowledge

We have made revisions throughout the manuscript, especially the Introduction, to differentiate this work from past work and better show how this paper contributes new knowledge.

- Limitations: The limitations presented raise serious concern over the findings and their value for reporting. Is there a reason none of the identified issues were addressed?

We revised the limitations section to explain what we did to mitigate them.

3. Discussion and recommendations need strengthening. At present while immediate references to other work are presented, as already mentioned the value added of this study is not sufficiently convincing. Additionally, the practical aspect of what this study means in terms of its use by a wider community of policy-makers, practitioners and researchers the statements lack underpinning by evidence from the work conducted and possibly wider literature. These sections need strengthening and sharpening.

We made revisions to better explain the value added of this study and to better articulate the practical usefulness of this study to a wider community.

II - Specific feedback:
- L51-56: Many off the tools reviewed here date pre 2018, therefore it is not surprising that the indicators are still missing in registers

We added language to explain that only 2 of the countries (Mozambique and Tanzania) have not updated any tools since the 2018 review.

- Table 1: Source / Reference to be included.

Added

- L109-113: A clear statement on how this work differs from what has been done previously is necessary, especially to flag value added of this study.

We have revised the discussion of other studies.

- L130-131: Why not? We added explanation for why we could not obtain certail tools into the text.

- L133-135: Why not? We added explanation for why we could not obtain certail tools into the text.

- L287-288: Is this recommendation valid in those countries, ie. is there a high rate of teenage pregnancies that would warrant this split being used? Please expand on this.

We added information on adolescent pregnancy in the context of countries in this review.

- L297-301: What is the insight from the countries regarding these points? Would it be possible to gather some inputs form the partners to provide more depth to the argument? Or seek more insight form wider literature?

We added information to explan these insights were based on real experience with existing tools.

- L301-304: This is a fair point, but needs to be substantiated to make it more robust. Are you suggesting the other countries do not have this sort of guidance?

We have clarified that not all countries had this sort of guidance.

- L307-308: This makes the findings somewhat shaky!

We revised the limitations section to explain what we did to mitigate the issue so it would have less impact on the findings.

- L308-309: Again, this is a serious issue when reporting!

See prior comment

- L309-312: Once more this is really big limitation!

See prior comment

- L312-316: As is this!

See prior comment

- L323-325: This is a suggestion that may require more insight - what is the current state of affairs in terms of standardisation of indicators used based on your review? What would wider literature? Please explain to provide more depth.

We revised the conclusion paragraph to provide more depth.

- Box 2: Were all countries studied in a similar manner to understand issues related to PPFP reporting? This is the level of detail required to understand the topic better.

Box 2 is meant to be an example of issues that arise as each country has a unique history and circumstance with regards to data systems, and it is not possible to analyze all in this paper.

III - Formatting, typographical errors and language:
- Author list: please check all names are separated by a comma and amend the font to make it consistent;

Checked

- All abbreviations need to be introduced at first use – please check and amend where needed (e.g. see LAM in Box 1);

Fixed

- Data: please standardise is singular or plus verb form is to be used and amend as required;

Has been checked and fixed where needed.

- L77: ‘during’ not ‘curing’ – please amend; Fixed

- The term 'delivery' has been updated to 'childbirth' - please consider revising to meet the current standards;

We replaced 'delivery' with 'childbirth' where appropriate.

- Tenses: consistency required (e.g. see L122-123);

These lines have been revised.

- Table 2: formatting of the table to be checked for consistency.

Formatting checked.
